# Effects of Different Orthoses on Neuromuscular Activity of Superficial and Deep Shoulder Muscles during Activities of Daily Living and Physiotherapeutic Exercises in Healthy Participants

**DOI:** 10.3390/jpm12122068

**Published:** 2022-12-15

**Authors:** Casper Grim, Christian Baumgart, Manuel Schlarmann, Thilo Hotfiel, Sasha Javanmardi, Natalie Hoffmann, Eduard Kurz, Jürgen Freiwald, Martin Engelhardt, Matthias W. Hoppe

**Affiliations:** 1 Center for Musculoskeletal Surgery Osnabrück (OZMC), Klinikum Osnabrück, Am Finkenhügel 1, 49076 Osnabrueck, Germany; 2 Institute for Health Research and Education (IGB), Faculty of Human Sciences, University of Osnabrueck, Nelson-Mandela-Straße 11, 49076 Osnabrueck, Germany; 3 Department of Movement and Training Science, University of Wuppertal, Gaußstraße 20, 42119 Wuppertal, Germany; 4 Institute of Human Movement and Sport Scienes, University of Osnabrueck, Jahnstraße 75, 49080 Osnabrueck, Germany; 5 Department of Trauma and Orthopaedic Surgery, University Hospital Erlangen, Friedrich-Alexander-Universität Erlangen-Nürnberg, Krankenhausstraße 12, 91054 Erlangen, Germany; 6 Department of Orthopedic and Trauma Surgery, Faculty of Medicine, Martin-Luther University Halle-Wittenberg, Ernst-Grube-Straße 40, 06112 Halle (Saale), Germany; 7 Movement and Training Science, Leipzig University, Jahnallee 59, 04109 Leipzig, Germany

**Keywords:** conservative therapy, CPM, EMG, mobilization, musculoskeletal complains, prevention, rehabilitation, rotator cuff, tendon, upper extremity

## Abstract

Background: This study aimed to investigate the effects of different shoulder orthoses on the neuromuscular activity of superficial and deep shoulder muscles during activities of daily living (ADL) and physiotherapeutic exercises. Methods: Ten participants with healthy shoulders (31 ± 3 years, 23.1 ± 3.8 kg/m^2^) were randomized to receive a “shoulder sling”, an “abduction pillow” and a “variably adjustable orthosis” on the dominant side. With each orthosis, they completed seven ADL with and four physiotherapeutic exercises without wearing the orthoses. An electromyographic system was used to record the neuromuscular activity of three superficial (trapezius, deltoid, pectoralis major) and two deep shoulder muscles (infraspinatus, supraspinatus) using surface and intramuscular fine-wire electrodes. Results: The neuromuscular activity differs between the orthoses during ADL (*p* ≤ 0.045), whereby the “variably adjustable orthosis” mostly showed the highest activation levels associated with the worst subjective wearing comfort rated on a visual analog scale. In addition, differences exist between the physiotherapeutic exercises (*p* ≤ 0.006) demonstrating the highest activations of the infra- and supraspinatus muscles for assistive elevation and wipe across a table, middle for pendulum and lowest for continuous passive motion exercises. Conclusions: The neuromuscular activity of superficial and deep shoulder muscles differs between the orthoses during ADL and also between the physiotherapeutic exercises.

## 1. Introduction

Immobilization is a fundamental part of conservative therapy and postoperative rehabilitation of various diseases and injuries of the shoulder joint and its girdle [1,2]. Over the last 20 years, orthotics has developed into a leading field of medical products [3,4]. However, the basic idea remained the same: partial immobilization, ensuring a prescribed joint position, and thereby stabilization and relief of the injured or surgically treated structures [1,5]. Additionally, ideas such as early functional treatment approaches and pathology-adapted immobilization positions were implemented over time [1]. Nowadays, it is well established to treat various acute and overuse injuries of the shoulder with different orthosis concepts, either as a primary conservative therapy option or part of the postoperative rehabilitation [1,3,6].

For a shoulder orthosis, different requirements can be defined. Predominantly, its use should lead to sufficient immobilization and reduction of neuromuscular activity to protect the injured or repaired tissue [5,7,8,9,10]. Another aspect is related to the early functional treatment, namely that it should be easy to take the orthosis on and off and allow the shoulder to be exercised by simply opening few fasteners or straps [5,9]. Additionally, it is important that sufficient mobility for activities of daily living (ADL) is retained [5]. It is self-evident that this includes not only tasks such as personal hygiene and dressing, but also independently putting on and taking off an orthosis. During such activities, it is known that the wearing comfort of an orthosis has an influence on the patient compliance [1]. In shoulder orthotics, three different retention and construction concepts can be distinguished: (i) sling-like orthosis with no abduction and mainly internally rotated position (hand on the belly), (ii) soft shoulder orthosis (abduction pillow) with an abducted position and a slightly variable rotation component and (iii) variable adjustable orthosis (more rigid construction) with an abduction and more fixed rotation position [1,6].

Postoperative rehabilitation protocols of the shoulder aim to balance between the deconditioning and stiffness of the joint that can be related to immobilization and harmful over-activity effects [6,7,8,9,10]. In fact, excessive neuromuscular activation of the rotator cuff muscles after surgery can contribute to a retearing of the reconstructed tendons [6,11,12]. Furthermore, an increased joint stiffness has also been shown to have adverse effects on clinical outcome and recovery [8]. Therefore, several postoperative protocols have been designed to retain joint mobility while safely loading the repaired tendons, including passive mobilization (e.g., continuous passive motion; CPM) and more active physiotherapeutic exercises (e.g., pendulum exercises) [5,6].

Since it is clear that superficial and deep shoulder muscles are simultaneously activated or coactivated during some motor tasks [13,14], it is of special interest to concurrently investigate the neuromuscular activity of both different muscle layers for a comprehensive evaluation of orthoses during ADL and physiotherapeutic exercises. Previous studies investigated either superficial or deep shoulder muscle activations during ADL or physiotherapeutic exercises including CPM via different electromyographic (EMG) approaches [15,16,17]. Moreover, only one previous study evaluated the effects of a not commercially available orthosis on the neuromuscular activity of shoulder muscles [18]. To the best of our knowledge, there is no study taking ADL and physiotherapeutic exercises into account by a comprehensive EMG-approach while also comparing the effects of different commercial orthoses with each other. Such knowledge may help to optimize shoulder orthoses and rehabilitation protocols.

The aim of the study was to investigate the effects of different shoulder orthoses on the neuromuscular activity of superficial and deep shoulder muscles during ADL. Additionally, the neuromuscular activity was examined during different physiotherapeutic exercises without wearing the orthoses. It was hypothesized that the neuromuscular activity of shoulder muscles differs between the orthoses during ADL and also between the physiotherapeutic exercises.

## 2. Materials and Methods

### 2.1. Participants

Five female and five male participants were recruited. The inclusion criteria were: (i) 18–35 years old, (ii) body mass index < 28.0 kg/m^2^, (iii) right-handed and (iv) signed written informed consent to participate. The exclusion criteria were: (i) previous or acute injuries, complaints or misalignments at the cervical and thoracic spine or shoulder joint, (ii) cognitive or psychological impairments, (iii) intake of blood thinners and (iv) neuromuscular diseases. The participants were informed about the purposes, procedures and potential risks of the study. All procedures were pre-approved and accepted by the Ethics Committee of the University of Wuppertal (MS/BBL; 190702) and were conducted in accordance with the Declaration of Helsinki. Table 1 summarizes the anthropometric characteristics of the participants.

### 2.2. Design

In this observational-experimental study, the data collection took place under standardized conditions in an examination room of a hospital over a 14-day period. Per each participant, the data collection lasted 180 min and was carried out on one day. After the participants had signed the written informed consent, a certified physiotherapist investigated the musculoskeletal system by routine clinical examinations for any exclusion criteria described above. Additionally, a specialist in orthopedics and trauma surgery examined the shoulder joint by a sonography device with a standardized preset. In case the participants could be included in the study, they were randomized to receive three different shoulder orthoses on the dominant right side. With each orthosis, the participants completed seven different ADL. Additionally, they performed four different physiotherapeutic exercises without wearing the orthoses. The ADL and physiotherapeutic exercises were defined due to their common occurrence and practical application as well according to previous studies [18,19,20]. During all movement tasks, an EMG-system was used to record the neuromuscular activity of selected superficial and deep shoulder muscles. In addition, an inertial measurement unit (IMU), a video camera and a visual analogue scale (VAS) were used to quantify the level of immobilization, beginning and end of each movement task and wearing comfort of each orthosis, respectively.

### 2.3. Shoulder Orthoses

**Figure 1:**
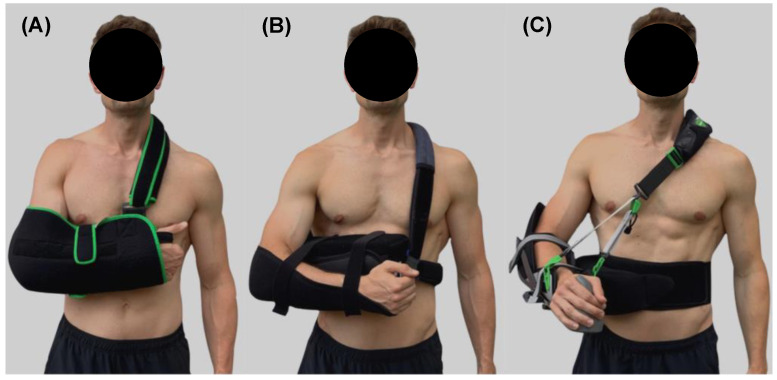
Applied shoulder orthoses. Note: (**A**) Orthosis #1 (shoulder sling); (**B**) Orthosis #2 (abduction pillow); (**C**) Orthosis #3 (variably adjustable orthosis).

Figure 1 shows the three used shoulder orthoses. These orthoses were selected due to their commercial availability, frequent application after surgical care and completely different immobilization concepts. Orthosis #1 was a “shoulder sling” that positioned the arm in internal rotation in front of the body and 0° abduction. Orthosis #2 was an “abduction pillow” with 10° internal rotation and 20° abduction; Orthosis #3 was a “variably adjustable orthosis” with neutral rotation position and 20° abduction. All orthoses were fitted according to the manufacturer’s instructions and under supervision of the physiotherapist.

### 2.4. Activities of Daily Living

With each orthosis, the participants performed seven different ADL in the following order:

#### 2.4.1. Stand Up-Sit Down from a Chair

The participants stood in front of a 50 cm high treatment couch, which corresponds to a seat high of many commonly used chairs in daily living. They sat down at their own pace, remained seated for 3 s and stood back up.

#### 2.4.2. Open-Close a Water Bottle

The participants stood in front of a 90 cm high worktop on which a bottle was placed. They were asked to grab the bottle with their left and open-close the bottle with their right hand. Then, they removed both hands from the bottle and remained standing for 3 s.

#### 2.4.3. Type the Alphabet on a Computer Keyboard

The participants sat on a 50 cm high chair in front of a 75 cm high desk. A standard computer keyboard was placed 12 cm from the edge of the table. Both hands were on the table. Then, they were asked to type the alphabet from A to Z using both hands. Upon completion, they took both hands off the table and remained seated for 3 s.

#### 2.4.4. Put On-Take Off a Jacket

In a standing position, the participants took their jacket from the back of a chair immediately to their left side. The jacket was considered to be put on, when the left arm was completely passed through the jacket sleeve and the jacket was placed over the right shoulder. Then, the participants remained standing for 3 s and took the jacket back off.

#### 2.4.5. Lie Down-Stand Up on a Couch

The participants stood in front of a 50 cm high treatment couch. The headboard was elevated by 10°. They were asked to lie down completely on their back, remain there for 3 s and stand back up.

#### 2.4.6. Walk Slowly over a Short Distance

The participants were asked to walk a 10 m distance on level ground at a self-chosen pace, remain standing for 3 s and walk the same distance back again.

#### 2.4.7. Put On-Take Off an Orthosis

According to the manufacturer’s instructions, the participants removed the orthosis independently and placed it on a chair immediately to their left side. Then, they remained in place for 3 s and put the orthosis back on.

### 2.5. Physiotherapeutic Exercises

Additionally, the participants completed the four different physiotherapeutic exercises without an orthosis accordingly.

#### 2.5.1. Wipe across a Table

The participants sat on a 50 cm high chair. Both hands lay folded on a towel placed on a 75 cm high table. The participants pushed the towel forwards to a mark placed 60 cm from the edge of the table. The left arm should guide the movement and the right shoulder should remain relaxed.

#### 2.5.2. Pendulum

The participants stood next to a 75 cm high table and placed their left hand so far forward on the table that their upper body was bent forward by 50°. From this starting position, the right arm was allowed to hang relaxed and swing loosely back and forth 10 times. Due to the relatively short duration of the movement, the participants performed the swinging 3 times.

#### 2.5.3. Assistive Elevation

The participants grabbed their right wrist with the left hand and moved the right arm upwards to 130° elevation of the shoulder joint. They were instructed to activate their right shoulder muscles as little as possible. Passive elevation was instructed and supervised during the measurements by a physiotherapist.

#### 2.5.4. Continuous Passive Motion

Finally, the participants sat on a CPM-chair. The chair was set to an initial position of 25° abduction. The right arm was passively moved until 60° abduction and then back to the initial position. The exercise was set to a duration of 5 min with an angular velocity of 90° per min.

Each movement task of the ADL and physiotherapeutic exercises was performed under the supervision of the physiotherapist 10 times and was separated by a 3 s rest period with exception of the pendulum und CPM exercises.

### 2.6. Data Collection

The collection of the EMG-, IMU-, video- and VAS-data was as follows:

#### 2.6.1. Electromyography

**Figure 2:**
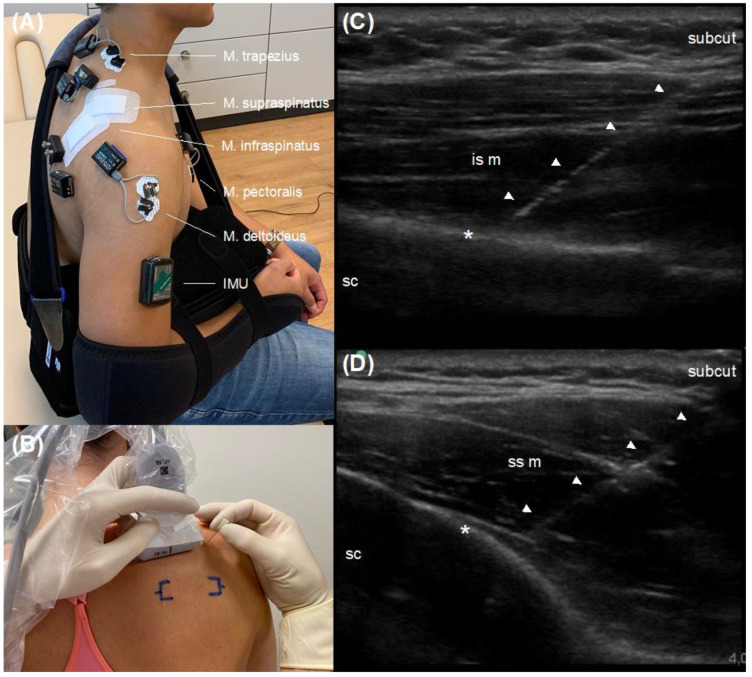
Placement of the electromyography electrodes on the investigated shoulder muscles. The placement of the inertial measurement unit is also shown. Note: (**A**) placement of electrodes and the inertial measurement unit (IMU); (**B**) sonography-guided application of the fine-wire electrodes; (**C**) positioning of electrode in the infraspinatus muscle; (**D**) positioning of electrode in the supraspinatus muscle. Abbreviations: subcut = subcutaneous fat tissue; is m = infraspinatus muscle; sc = scapula; ss m = supraspinatus muscle; asterisk = cortical interface; arrows = intramuscular needle position.

A telemetric EMG-system (NORAXON, Clinical DTS, Scottsdale, AZ, USA) was used to record the neuromuscular activity of three superficial and two deep shoulder muscles. As superficial muscles, the trapezius (pars descendens), deltoid (pars acromialis) and pectoralis major (pars sternalis) muscles were captured using surface electrodes (HEX Dual Electrodes, NORAXON, Scottsdale, AZ, USA) in a bipolar configuration. The arrangement of the electrodes and skin preparation were conducted according to established guidelines [21,22]. Additionally, deep shoulder muscles of the rotator cuff, namely the infraspinatus and supraspinatus muscles, were examined using intramuscular fine-wire electrodes. To ensure an accurate placement of the electrodes [23], an ultrasound device (M-Turbo, Fujifilm Sonosite, Amsterdam, The Netherlands) was utilized for a real-time sonography-guided percutaneous application. The applications were performed by a specialist in orthopedics and trauma surgery, who is skilled and certified according to the German Society of Ultrasound in Medicine (DEGUM). The intrusion depth was set at 40 mm and the focus was centered at 50% cross-sectional muscle diameter. A linear probe (HFL50x, Sonosite, Amsterdam, The Netherlands) with a frequency of 12 MHz within standardized musculoskeletal preset was positioned at the posterior shoulder aspect in longitudinal axis for the infraspinatus and supraspinatus muscle separately. A 75 mm needle (Chalgren Enterprises, Gilroy, CA, USA) was then aligned via in-plane technique visualizing the entire shaft and needle tip. When an accurate position was achieved, the needle was released leaving the fine-wire electrodes intramuscularly centered. The procedure was digitally recorded for each participant. All procedures were performed under sterile standards according to established recommendations [24]. Figure 2 shows the placement of the EMG-electrodes to the investigated shoulder muscles.

For each of the shoulder muscles, a maximal voluntary contraction (MVC) test was conducted to normalize the recorded EMG-data. Therefore, ramped maximal isometric muscle contractions over 5 s in duration were performed and repeated after a 60 s recovery period 3 times. The particular procedure for each muscle was as follows: The trapezius (pars descendens) muscle was activated caudally by giving resistance of the physiotherapist from above on the shoulder. Then, the participants were instructed to pull the shoulder upwards. For the pectoralis major (pars sternalis) muscle, the participants were instructed to grasp a rod with both hands a shoulder-width apart and hold it at shoulder height in front of the upper body. The physiotherapist stood behind the participants and applied resistance by pulling the rod dorsally. The participants were instructed to push the rod away from themself. For the supraspinatus and deltoideus muscles, the upper arm was in zero position in the shoulder joint with the forearm flexed by 90° to control the neutral rotational position of the shoulder joint. Then, the upper arm was tensed against resistance inducted by the physiotherapist at the distal upper arm in abduction. For the infraspinatus muscle, the upper arm was tensed in external rotation at 90° from zero position on the shoulder joint. To prevent abduction, the participants were instructed to fix the left hand of the physiotherapist to their upper body with their elbows.

All EMG amplifiers and cables were fixed by double-sided adhesive tape and sterile patches (Figure 2). The EMG-data were acquired at 1500 Hz, processed using standard procedures (25 Hz Butterworth high-pass filter, 200 ms RMS) and MVC-normalized (500 ms) over all trials as mean activation per movement [25,26].

#### 2.6.2. Inertial Measurement Unit

An IMU-device (NORAXON, myoMOTION Research Pro, Arizona, USA), collecting 3D accelerometer data at 100 Hz, was used to quantify the total amount of immobilization of the shoulder joint, due to both active muscle contraction and passive external caused movements, induced by the different orthoses. The unit was placed by double-sided adhesive tape on the middle distance of the upper arm (Figure 2). To quantify the global movement of the upper arm during the ADL, the numerical integral of the resulting acceleration was calculated and averaged over all trials [27].

#### 2.6.3. Video Analysis

A video camera (Logitech, C920; Lausanne, Switzerland) operating at 30 Hz was used to detect the beginning and end of each movement task within the recorded EMG- and IMU-data. A lamp was placed in the recorded video for synchronizing.

#### 2.6.4. Visual Analogue Scale

A 10 cm VAS was used to rate the wearing comfort of each orthosis. The ratings were conducted by the participants after all devices and materials have been removed from the body.

### 2.7. Statistical Analysis

All data were collected and proceeded by manufacturer software (NORAXON, MyoResearch 3.18, Arizona USA). The statistical analysis was performed in RStudio (vers. 2022.7.1.554) [28,29]. Due to the non-given normal distribution of several data and small sample size, non-parametric statistical tests were applied. Friedman tests were used to detect significant mean effects between the three orthoses during ADL and between the four physiotherapeutic exercises. In terms of global significance, Wilcoxon-Signed-Rank tests were used for post-hoc analyses. Corresponding effects sizes according to Kedall’s W were computed and interpreted accordingly: 0.1 to <0.3, small; 0.3 to <0.5, moderate and ≥0.5, large [30]. The threshold for global statistical significance was set at *p* < 0.05, whereas Holm corrections were applied to control for type 1 errors during post-hoc testing.

## 3. Results

### 3.1. Effects of Orthoses on Neuromuscular Activity during Activities of Daily Living

**Figure 3:**
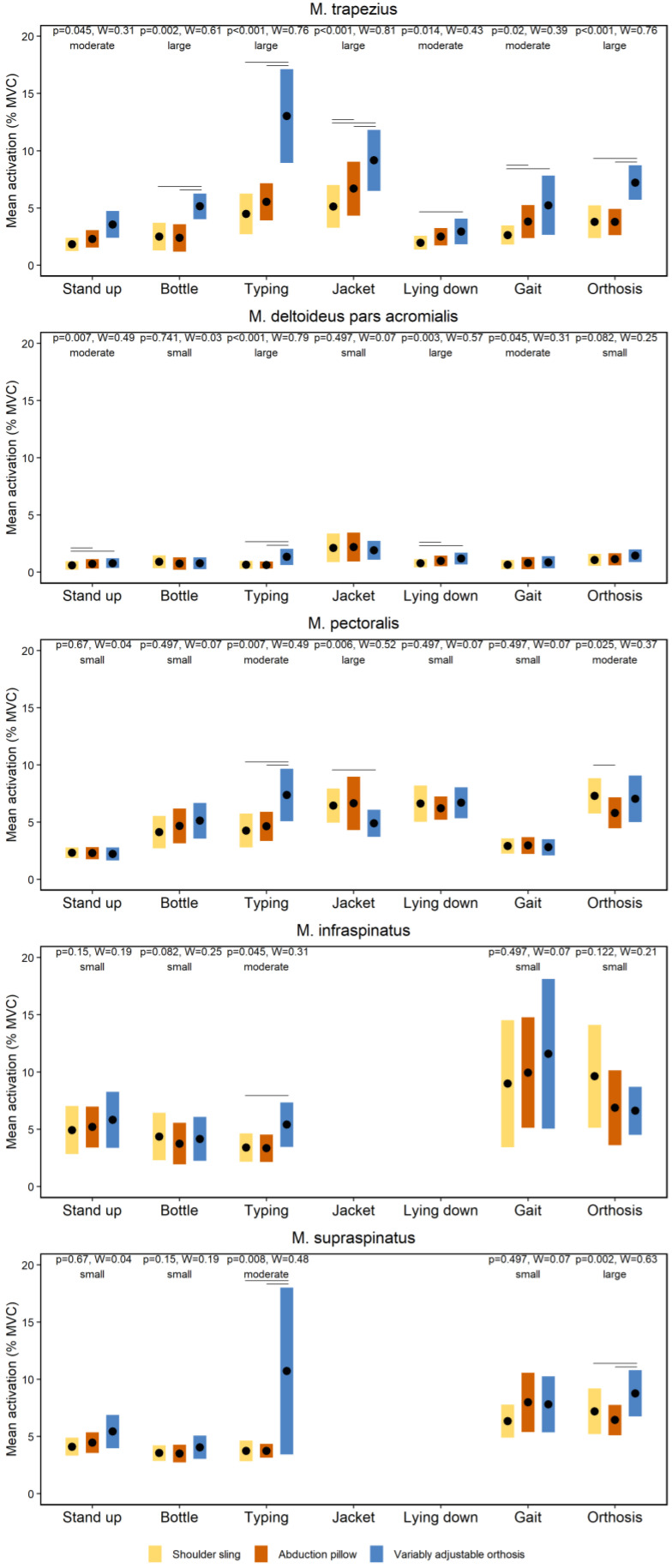
Effects of the orthoses on the neuromuscular activity of the shoulder muscles during the activities of daily living. Note: Diamonds and bars indicate mean activations (%MVC) and 90% confidence intervals, respectively. Horizontal lines indicate significant post-hoc effects. Data of the infraspinatus and supraspinatus muscles during two movement tasks (jacket and lying down) could not be analyzed due to movement artefacts within the fine-wire electrodes.

Figure 3 shows the effects of the orthoses on the neuromuscular activity of the shoulder muscles during the ADL. The neuromuscular activity of shoulder muscles differs between the selected orthoses during ADL. Particularly, the variably adjustable orthosis (orthosis #3) showed the highest activation levels (Figure 3). The Friedman tests revealed several global significant effects (*p* ≤ 0.045). The corresponding effect sizes ranged from moderate to large (W ≥ 0.31). The Wilcoxon-Signed-Rank tests showed numerous significant post-hoc effects summarized in Figure 3.

**Figure 4:**
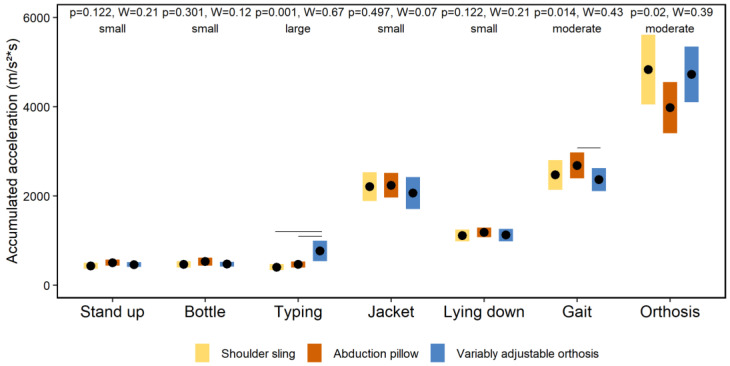
Effects of the orthoses on the total amount of immobilization of the shoulder joint during the activities of daily living. Note: Diamonds and bars indicate accumulated accelerations and 90% confidence intervals, respectively. Horizontal lines indicate significant post-hoc effects.

**Figure 5:**
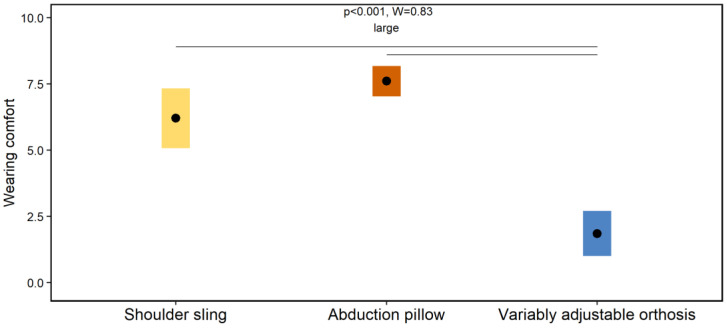
Effects of the orthoses on the wearing comfort during the activities of daily living. Note: Diamonds and bars indicate mean ratings and 90% confidence intervals, respectively. Horizontal lines indicate significant post-hoc effects.

Figure 4 and Figure 5 show the effects of the orthoses on the total amount of immobilization of the shoulder joint and wearing comfort during the ADL, respectively. The Friedman tests showed several global significant effects on the immobilization (*p* ≤ 0.02) and wearing comfort (*p* < 0.001). The associated effect size(s) ranged from moderate to large (W ≥ 0.39). The Wilcoxon-Signed-Rank tests showed numerous significant post-hoc effects summarized in Figure 4 and Figure 5.

### 3.2. Effects of Physiotherapeutic Exercises on Neuromuscular Activity

**Figure 6:**
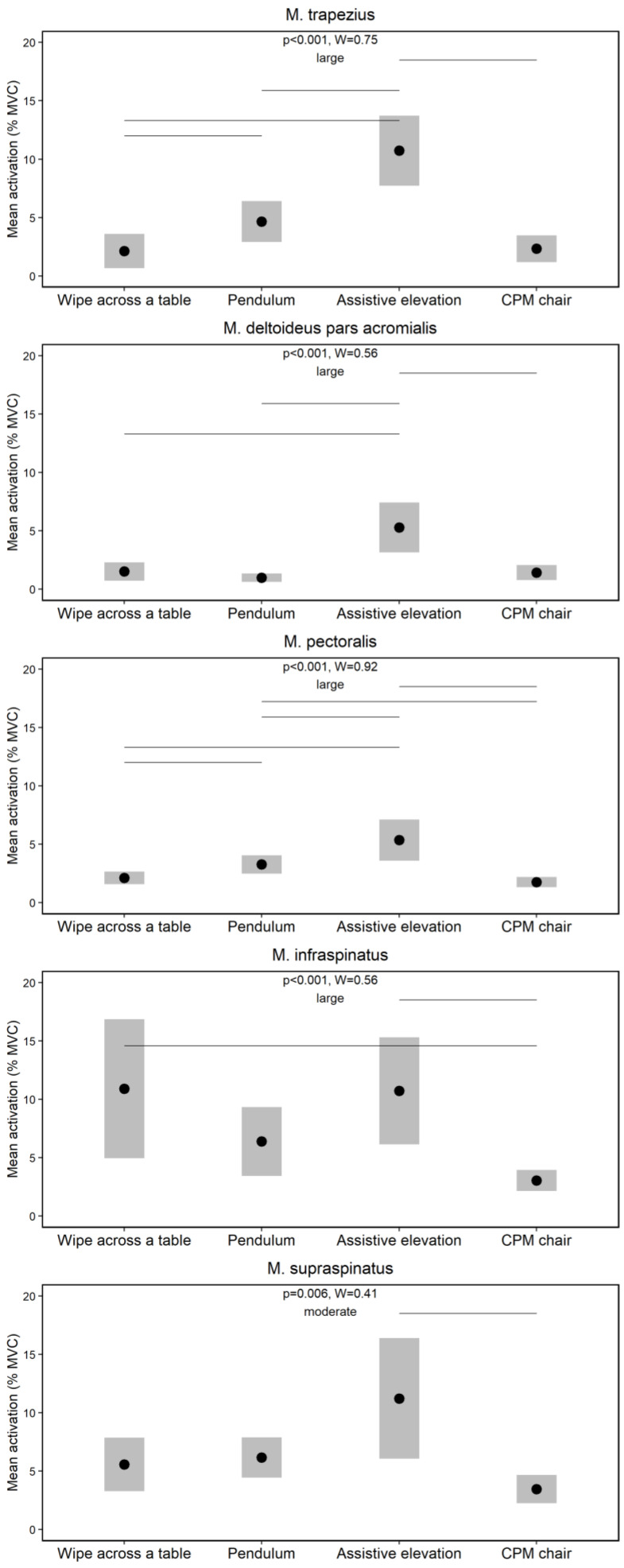
Effects of the physiotherapeutic exercises on the neuromuscular activity of the shoulder muscles. Note: Diamonds and bars indicate mean activations (%MVC) and 90% confidence intervals, respectively. Horizontal lines indicate significant post-hoc effects.

Figure 6 shows the effects of the physiotherapeutic exercises on the neuromuscular activity of the shoulder muscles. The Friedman tests demonstrated several global significant effects (*p* ≤ 0.006) supported by moderate to large effect sizes (W ≥ 0.41). The Wilcoxon-Signed-Rank tests indicated numerous significant post-hoc effects summarized in Figure 6.

## 4. Discussion

This study investigated the effects of three different orthoses on the neuromuscular activity of superficial and deep shoulder muscles during ADL. Additionally, the neuromuscular activity was examined during four different physiotherapeutic exercises without wearing the orthoses. Our main findings were that the neuromuscular activity of shoulder muscles differ between (i) the orthoses during ADL, whereby the variably adjustable orthosis mostly showed highest activation levels; and between (ii) the physiotherapeutic exercises demonstrating highest activations for the assistive elevation and wipe across a table exercises, especially of the infra- and supraspinatus muscles.

The first main finding was that the neuromuscular activity of shoulder muscles differs between the selected orthoses during ADL. Particularly, the variably adjustable orthosis (orthosis #3) showed the highest activation levels (Figure 3). A previous study shows that neuromuscular rotator cuff activity of healthy participants did not exceed 11% MVC during ADL while wearing an orthosis [18], which is similar to the MVC-data of this study (Figure 3) and supports our methodological approach. However, compared to the previous study [18], all of our investigated shoulder orthoses are commercially available, allowing more external valid conclusions for clinical care. In our study, it was hypothesized that the different immobilization concepts of the orthoses (Figure 1) lead to different neuromuscular activations of the shoulder muscles during ADL. This could not be clearly confirmed by our results. Generally, the variable adjustable orthosis (orthosis #3) shows higher activations compared to the shoulder sling (orthosis #1) and abduction pillow (orthosis #2), whereby both latter orthoses mainly did not differ (Figure 3). One speculation for these findings may be that the neuromuscular activation of shoulder muscles is primarily not caused by the immobilization concepts of the orthoses, but rather by the perceived subjective wearing comfort [1]. This assumption is supported by both the findings of a previous study, showing that soft shoulder orthoses are perceived as more comfortable than harder ones [31] and our data. In fact, the total amount of immobilization of the shoulder joint induced by the different orthoses during ADL, as quantified by IMU-data, did overall not systematically differ (Figure 4). Instead, the variable adjustable orthosis (orthosis #3), constructed of harder material (Figure 1), showed a lower wearing comfort than both other orthoses (Figure 5), and this worst comfort was associated with the highest neuromuscular activation (Figure 3). Thus, the wearing comfort of a shoulder orthosis must be considered when interpreting its immobilization effect by measuring neuromuscular activation levels and is an essential aspect for patient compliance [1]. However, it is recommended that a neuromuscular activation of <15% MVC is required for a safe healing of the injured or surgery treated tissue during the shoulder rehabilitation [32,33]. From this perspective, all three shoulder orthoses can be considered as safe for ADL during the rehabilitation (Figure 3), requiring more research.

The second main finding was that the neuromuscular activity of shoulder muscles differs between the chosen physiotherapeutic exercises. Surprisingly, the highest activations were evident for the passive assistive elevation and wipe across a table exercises, especially of the infra- and supraspinatus muscles (Figure 6). A classification of our data with those of previous studies [15,19,33,34,35] is difficult due to differences in the selection and execution of exercises. However, a systematic review [32] identified several physiotherapeutic exercises that were considered to be appropriate during the early postoperative rehabilitation after surgery of the infra- and supraspinatus muscles due to their low neuromuscular activation (MVC < 15%). These included comparable wipe across a table, pendulum and assistive elevation exercises that were also examined in a comparable manner in our study (Figure 6), supporting their application during the rehabilitation and again our methodological approach. A new finding of our study was that also a commonly performed passive exercise during the early postoperative rehabilitation, namely a CPM-chair [6], was found to have a low neuromuscular activation (MVC < 15%) (Figure 6). A previous study compared the neuromuscular activity of shoulder muscles during different passive exercises only and found lowest activation levels for CMP [36], which is in line with our findings (Figure 6). Although a clear differentiation between active and passive rehabilitation exercises is difficult, especially in patients where protective muscle co-activations can be evident [14,15], the CPM-chair always showed lowest neuromuscular activation levels, which should be considered for the progression of exercise selection during the early postoperative rehabilitation [6]. Taken overall, the investigated physiotherapeutic exercises can be considered as safe due their low neuromuscular activation (MVC < 15%), whereas the highest activations of the infra- and supraspinatus muscles were found for the assistive elevation and wipe across a table, the middle for the pendulum, and the lowest for the CPM-chair. These hierarchical outcomes can be helpful for the periodization of rehabilitation protocols after surgical shoulder treatments, for which further investigations are essential.

An interesting additional finding was that the neuromuscular variability of deep was higher than those of superficial shoulder muscles. Predominantly, this higher variability was evident during the more dynamic movement tasks, such as walk slowly and put on-take off orthosis (Figure 3) as well as wipe across table and assistive elevation (Figure 6). Previous studies also showed higher neuromuscular variations for deep than superficial shoulder muscles during similar exercises [33,36], as indicated by higher dispersion data, which is in line with our calculations (Figure 3 and Figure 6). While the underlying reasons remain unknown, both methodological and physiological causes can be assumed. For example, and compared to non-invasive surface electrodes, potential fewer active motor units are derived by the intramuscular fine-wire approach [37], which may explain the higher variability from an EMG-methodological point of view. A physiological point can be related to potential perceived pains or discomforts inducted by the invasive approach that could lead to more spontaneous discharges of motor units [37]. One could also speculate about a less voluntary motor control of the deep compared to the superficial shoulder muscles, which has been discussed for other deep muscles such as the multifidi [38,39]. However, from an evolutionary perspective, the human shoulder muscles are extremity muscles [40,41] and have a different type of innervation compared to the multifidus muscles [42]. Thus, more research to clarify the observed higher variability of deep shoulder muscles is warranted. However, from a clinical perspective, our variability outcomes underline that an “one treatment fits all” approach is—of course—not timely in the present context. Instead, a personalized postoperative treatment approach concerning the selection of shoulder orthoses and physiotherapeutic exercises is obligatory.

While our study undoubtedly increased the knowledge on the neuromuscular activity of superficial and deep shoulder muscles during ADL while wearing different shoulder orthoses and during physiotherapeutic exercises performed without the orthoses, the main noteworthy limitation is related to our relatively young and shoulder healthy participants. Since it is clear that the shoulder biomechanics of injured or surgery treated patients is completely different to healthy participants [43], caution should be used in transferring our results into the medical care. When designing the study, we could not rule out that some settings may exceed a certain limitation of shoulder immobilization, so that patients might have been jeopardized. Based on our results and the defined measurement setup, further studies recruiting surgically treated patients should be implemented to relating data to clinical conditions. Future studies should focus on older patients with traumatic injuries, degenerative changes or generally on patients at post-surgical situations. We acknowledge that our measurements including healthy patients represent short-term effects of ADL and physiotherapeutic exercises on neuromuscular activity. A continuous application of orthosis or a long-term immobilization may lead to functional impairments and adaptions. Hence, our data are not transferable to long-term conditions. Follow-up investigations assessing changes of neuromuscular activity related to the long-term use of orthosis need to be conducted.

## 5. Conclusions

This study suggests that it is predominantly not the immobilization concept of shoulder orthoses, but rather the wearing comfort that supports a low neuromuscular activation of superficial and deep shoulder muscles during ADL. In regard to common physiotherapeutic exercises performed during the early postoperative rehabilitation, the highest activations of the infra- and supraspinatus muscles were found for the assistive elevation and wipe across a table, the middle for the pendulum, and the lowest for the CPM-chair. Our results could be used to guide the orthosis selection and type of physiotherapeutic exercises. We are convinced that our results can provide useful information for both orthosis designers and clinicians.

## Figures and Tables

**Table 1:** Anthropometric characteristics of the participants.

Variable	Mean ± SD
Age (years)	31± 3
Body height (m)	1.72 ± 0.08
Body mass (kg)	68.9 ± 14.8
Body mass index (kg/m^2^)	23.1 ± 3.8

## Data Availability

The datasets used and analyzed during the current study are available from the corresponding author on reasonable request.
